# Differential mechanisms of asparaginase resistance in B-type acute lymphoblastic leukemia and malignant natural killer cell lines

**DOI:** 10.1038/srep08068

**Published:** 2015-01-28

**Authors:** Wei-Wen Chien, Céline Le Beux, Nicolas Rachinel, Michel Julien, Claire-Emmanuelle Lacroix, Soraya Allas, Pierre Sahakian, Aurélie Cornut-Thibaut, Loïc Lionnard, Jérôme Kucharczak, Abdel Aouacheria, Thierry Abribat, Gilles Salles

**Affiliations:** 1 Université Claude Bernard Lyon 1, UMR 5239 CNRS ENS HCL, Faculté de Médecine Lyon Sud, 165 Chemin du Grand Revoyet, 69921, BP12, Oullins, FRANCE; 2 Alizeé Pharma, 15 Chemin du Saquin, Espace Européen, Building G, 69130, Ecully, FRANCE; 3 Hospices Civils de Lyon, Service d'Hématologie, 165 Chemin du Grand Revoyet, 69495 Pierre-Bénite, FRANCE

## Abstract

Bacterial L-asparaginase (ASNase), hydrolyzing L-asparagine (Asn), is an important drug for treating patients with acute lymphoblastic leukaemia (ALL) and natural killer (NK) cell lymphoma. Although different native or pegylated ASNase-based chemotherapy are efficient, disease relapse is frequently observed, especially in adult patients. The neo-synthesis of Asn by asparagine synthetase (AsnS) following ASNase treatment, which involves the amino acid response and mitogen-activated protein kinase kinase/extracellular signal-regulated kinase pathways, is believed to be the basis of ASNase-resistance mechanisms. However, AsnS expression has not emerged as an accurate predictive factor for ASNase susceptibility. The aim of this study was to identify possible ASNase sensitivity/resistance-related genes or pathways using a new asparaginase, namely a pegylated r-crisantaspase, with a focus on classic Asn-compensatory responses and cell death under conditions of Asn/L-glutamine limitation. We show that, for B-ALL cell lines, changes in the expression of apoptosis-regulatory genes (especially NFκB-related genes) are associated with ASNase susceptibility. The response of malignant NK cell lines to ASNase may depend on Asn-compensatory mechanisms and other cellular processes such as cleavage of BCL2A1, a prosurvival member of the Bcl-2 protein family. These results suggest that according to cellular context, factors other than AsnS can influence ASNase susceptibility.

Bacterial L-asparaginase (ASNase) is an important component of anticancer regimens for acute lymphoblastic leukemia (ALL)^1^,^2^. Recently, there has also been a renewed interest in the use of ASNase in non-Hodgkin lymphoma treatment^2^, especially in extranodal natural killer (NK)/T-cell lymphoma patients^3^,^4^,^5^. ASNase catalyzes the hydrolysis of L-asparagine (Asn). Its anti-leukemic effect is associated with the ability to deplete quickly and completely the pool of Asn in the circulating blood and bone marrow, leading to subsequent depletion of Asn in leukemic cells^2^,^6^,^7^. It has generally been considered that the higher susceptibility of leukemic cells to ASNase is related to their low expression of Asn synthetase (AsnS), compared to non-transformed B-cells^8^,^9^,^10^,^11^. In addition, the minor L-glutaminase (GLNase) activity of ASNase, which catalyzes the hydrolysis of L-glutamine (Gln) (the only nitrogen donor for Asn synthesis), may also participate to the anti-leukemic action of ASNase^12^,^13^,^14^,^15^.

Despite the high survival rate of patients treated with chemotherapeutic drugs including ASNase, about 20% of children and more than 50% of adults with ALL relapse, with leukemic cells becoming resistant to treatment^1^. Researches, using multi-step selected human cancer cell lines resistant to ASNase, have demonstrated that many adaptive changes occur in ASNase-resistant cells, including: 1) an increase in *AsnS* mRNA and protein expression; 2) a decrease in Asn efflux through Na^+^-independent exchange system; 3) a production of aspartic acid, the substrate for Asn synthesis by AsnS, via transamination; 4) a higher Gln synthetase (GlnS) activity through post-transcriptional regulation; 5) an activation of the Gln transporter^9^,^10^,^11^,^16^,^17^,^18^. In addition, inhibition of AsnS or GlnS expression or activity can sensitize resistant cells to ASNase^13^,^14^,^19^,^20^,^21^.

Moreover, at the level of signaling transduction, the amino acid response (AAR) pathway, a well-known pathway that senses and responses to a deficiency of amino acids, as well as the survival-related mitogen-activated protein kinase kinase (MEK)/extracellular signal-regulated kinase (ERK) and mammalian target of rapamycin complex 1 (mTORC1) pathways are believed to be involved in ASNase resistance through supplying Asn and limiting its waste. Indeed, upon amino acid depletion, uncharged tRNAs activate general control nonderepressible 2 kinase (GCN2) of AAR pathway, which phosphorylates subsequently eukaryotic initiation factor 2 alpha (elF2α), leading to translational up-regulation of a transcription factor ATF4. Enriched ATF4 proteins promote activation of *AsnS* transcription for further neo-synthesis of Asn^10^,^22^,^23^,^24^,^25^,^26^,^27^. The fact that a MEK/ERK inhibitor blocks the activation of AAR pathway and that enhanced phosphorylation of Erk1/2 also requires GCN2 kinase activity, suggests interdependence between the AAR and MEK/ERK pathways in response to amino acid deficiency^28^. The activity of the mTORC1 complex is inhibited by amino acid restriction, as indicated by dephosphorylation of downstream factors 4E-BP1 and ribosomal protein S6K, preventing Asn consuming in futile translation of mRNA^27^,^29^,^30^,^31^. Inhibition of the mTORC1 pathway occurring downstream of GCN2 activation has been suggested since three transcriptional targets of ATF4, GADD34, 4E-BP1 and REDD1, may negatively regulate the mTORC1 signaling^32^,^33^. It has also been reported that phosphorylation of mTORC1 targets (4E-BP1 and S6K) is reduced, in a GCN2-dependent manner^34^, in the liver and pancreas of mice treated with ASNase.

Although AsnS expression levels are believed to be a key factor in these mechanisms of resistance to ASNase, several studies using clinical samples from children and adult ALL fail to show any correlations between the sensitivity to ASNase and the mRNA expression of *AsnS* at baseline or after exposure to ASNase^35^,^36^,^37^,^38^. It has also been reported that the mRNA and protein expression of *AsnS* in ALL patient samples are generally so low that *AsnS* expression cannot be used as a predictive factor for ASNase sensitivity or resistance^37^. In addition, *AsnS* has not been identified as a discriminating gene for ASNase sensitivity in the study of gene-expression patterns in sensitive versus resistant children ALL samples^39^. Thus, the importance of AsnS in ASNase resistance has been challenged, pointing to the need to determine if other factors contribute to the resistance phenotype.

In the present work, we sought to identify discriminating factors for ASNase susceptibility using two pairs of B-ALL and malignant NK cell lines with high or low sensitivity to ASNase. We demonstrate that the classical resistance-mechanisms involving the MEK/ERK pathway as well as increase in GlnS expression are associated with resistance to ASNase in the KHYG1 NK leukemia cell line. Furthermore, we show that ASNase induced cell death by apoptosis and that the induction of apoptosis after exposure to ASNase is delayed in resistant cell lines. Analysis of gene-expression indicates that activation of the NFκB pathway is associated with higher sensitivity of the RS(4, 11) B-ALL cell line, while cleavage of the prosurvival Bcl-2 family member BCL2A1 was observed in the sensitive MEC04 NK T-cell lymphoma cell line. These data point to different mechanisms involved in ASNase-susceptibility according to disease type.

## Results

### Classic compensatory mechanisms are involved in the resistance of KHYG1 cells to ASNase

In our previous study, we have defined using MTT assay the susceptibility of different leukemic cell lines (including the two B-ALL cell lines RS(4, 11) and Nalm-6), non-Hodgkin lymphoma cell lines as well as malignant NK cell lines (including the NK T-cell lymphoma cell line MEC04 and the NK cell leukemia cell line KHYG1), to ASNase treatment^40^. RS(4, 11) and MEC04 cells were found to be very sensitive to ASNase (EC_50_ < 5 × 10^−5^), whereas Nalm-6 and KHYG1 were much less sensitive to ASNases (EC_50_ > 1 × 10^−2^)^40^. These two pairs of cell lines with low or high sensitivity to ASNase, which we term here ‘resistant’ or ‘sensitive’ for clarity, were selected for further analysis of the cellular response to ASNase.

**Figure 1:**
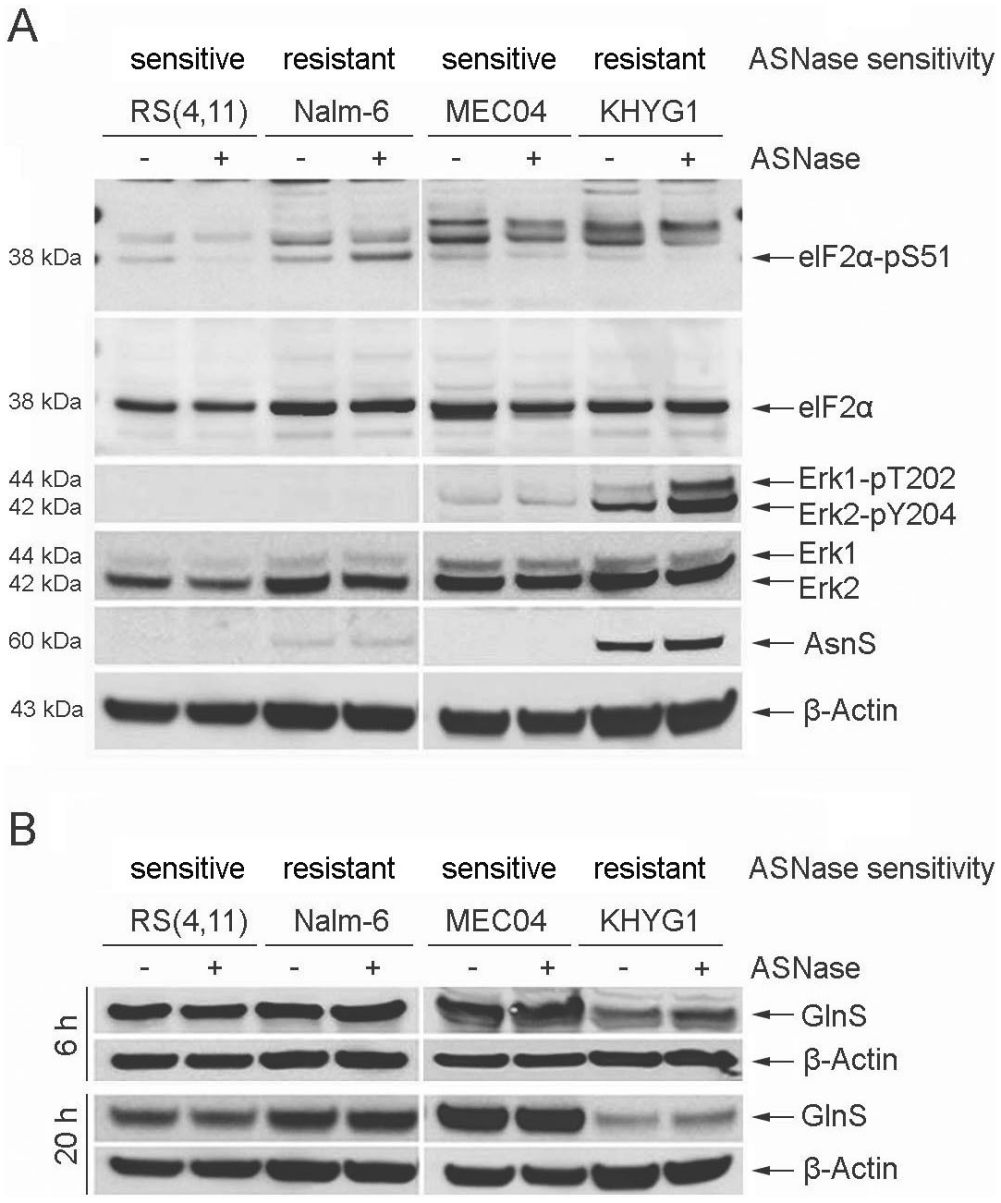
Expression of phosphorylated elF2α, phosphorylated Erk1/2, AsnS and GlnS following ASNase treatment. B-ALL (RS(4, 11), Nalm-6) and malignant NK (MEC04, KHYG1) cell lines were treated with 5 U/ml of pegylated-r-crisantaspase for 6 h or 20 h. Non-treated cells served as controls. Protein expression was analyzed by Western Blot. For the proteins elF2α and elF2α-pS51, all samples were loaded in the same gel. For other proteins, samples were loaded in different gels (one gel for RS(4, 11) and Nalm-6 cell lines and one gel for MEC04 and KHYG1 cell lines). Two gels have been run under the same experimental conditions. The experiment was repeated twice with similar results.

We first analyzed the classic compensatory AAR and MER/ERK pathways in the four cell lines (Figure 1A). Without ASNase treatment, the phosphorylation of elF2α on Ser51 was detectable in all four cell lines. The phosphorylation of Erk1/2 on T202/Y204 were absent in B-ALL cell lines but detectable in malignant NK cell lines. The expression of AsnS was undetectable in sensitive RS(4, 11) and MEC04 cell lines, whereas resistant Nalm-6 and KHYG1 cells expressed AsnS, although the expression was weak in Nalm-6 cells. Upon ASNase exposure for 20 h, elF2α phosphorylation was only increased in Nalm-6 cells, yet reduced in 3 other cell lines. Erk1/2 phosphorylation was greatly induced in KHYG1 cells, with AsnS expression slightly induced. In addition, we analyzed the expression of GlnS in these cell lines (Figure 1B), since the increase in Gln synthesis catalyzed by GlnS can influence the susceptibility of cells to ASNase. While abundant levels of GlnS were detected in RS(4, 11), Nalm-6 and MEC04 cell lines, this enzyme was less expressed in KHYG1 cells at baseline. Yet, only in this cell type, ASNase treatment induced a slight increase in the GlnS protein levels.

These results indicate that the increased activity of the MEK/ERK pathway and the moderate up-regulation of AsnS and GlnS expression, favoring the neo-synthesis of Asn to compensate for Asn limitation, are associated to the ASNase-resistance of KHYG1 cells.

### Decreased activity of the mTORC1 pathway after exposure to ASNase is a common feature of sensitive and resistant cells

**Figure 2:**
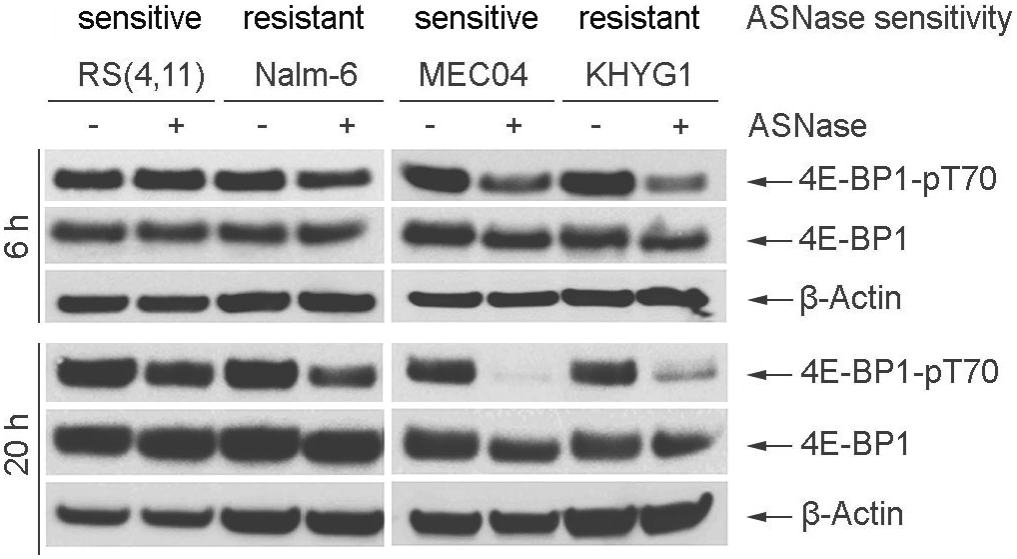
Expression of phosphorylated 4E-BP1 following ASNase treatment. B-ALL (RS(4, 11), Nalm-6) and malignant NK (MEC04, KHYG1) cell lines were treated with 5 U/ml of pegylated-r-crisantaspase for 6 h or 20 h. Non-treated cells served as controls. Protein expression was analyzed by Western Blot. Samples were loaded in different gels (one gel for RS(4, 11) and Nalm-6 cell lines and one gel for MEC04 and KHYG1 cell lines). Two gels have been run under the same experimental conditions. The experiment was repeated twice with similar results.

To determine whether differences in mTORC1 activity exist between sensitive and resistant cell lines, the phosphorylation of 4E-BP1 on T70 was analyzed (Figure 2). For malignant NK cell lines, a sharp decrease in the phosphorylation of 4E-BP1 was observed in both sensitive and resistant cell lines 20 h after exposure to ASNase, an approximately 2-fold decrease being evident as early as 6 h post-treatment. 4E-BP1 phosphorylation was almost undetectable in sensitive MEC04 cells 20 h post-treatment whereas a residual phosphorylation remained detectable in resistant KHYG1 cells. With regard to B-ALL cell lines, 4E-BP1 phosphorylation was slightly decreased in resistant Nalm-6 cells 6 h post-treatment, and increased in sensitive RS(4, 11) cells. An obvious reduction in 4E-BP1 phosphorylation in both cell lines was observed 20 h post-treatment.

These observations show that in response to ASNase treatment the expression of phosphorylated 4E-BP1 was reduced in both the sensitive and the resistant cell lines and that the activity of mTORC1 pathway does not represent a discriminating factor for ASNase susceptibility in these cell lines.

### Delayed induction of apoptosis in resistant cell lines in response to ASNase treatment

**Figure 3:**
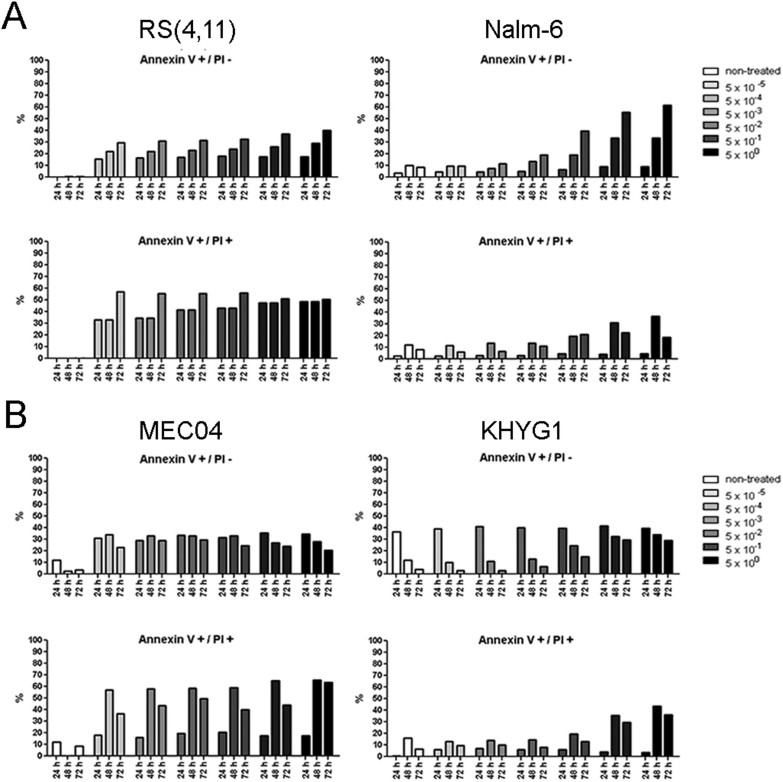
Analysis of cell death modality and timing after exposure to ASNase. B-ALL (RS(4, 11), Nalm-6) and malignant NK (MEC04, KHYG1) cell lines were treated with increasing doses of pegylated-r-crisantaspase (from 5 × 10^−5^ to 5 U/ml). Non-treated cells served as controls. The percentage of early apoptotic (Annexin V+/PI−) and late apoptotic (Annexin V+/PI+) was determined by flow cytometry after Annexin V/PI staining.

Next, we determined the cell death modality (apoptosis or necrosis) through which ASNase-treated cells were dying, and the difference in the induction of cell death between sensitive and resistant cell lines (Figure 3). For B-ALL cell lines, we observed an increase in the proportion of sensitive RS(4, 11) cells in early apoptosis (Annexin V+/PI- cells, ~15%) and late apoptosis (Annexin V+/PI+ cells, ~32%) 24 h after exposure to ASNase and this increase was detectable even at low doses (e.g. 5 × 10^−5^/ml). In contrast, the induction of apoptosis (~23%) and late apoptosis, (~18%) in resistant Nalm-6 cells was observed only 48 h after ASNase treatment and only at high doses of ASNase (e.g. ≥ 5 × 10^−1^ U/ml) (Figure 3A).We observed similar profiles of early/late apoptosis induction for the malignant NK sensitive (MEC04) and resistant (KHYG1) cell lines (Figure 3B). In all 4 studied cell lines, the proportion of necrotic (Annexin V−/PI+) cells was very low even 72 h after ASNase treatment (data not shown).

These results indicate that B-ALL and malignant NK cells predominantly die by apoptosis in response to ASNase treatment and that, expectedly, the induction of apoptosis is delayed and less marked in resistant versus sensitive cell lines.

### Differential expression of NFκB pathway-related factors at baseline and in response to ASNase

In order to determine which apoptosis-related factors may influence the susceptibility of cells to ASNase, the mRNA expression of 93 apoptosis-related genes was analyzed using TaqMan® Human Apoptosis Array in cells treated or not with ASNase. According to their prominent function in the regulation or execution of apoptosis, the studied genes were classified as pro-apoptotic (n = 72) or anti-apoptotic (n = 21) and were divided into four sub-groups: Bcl-2 family, caspases, NFκB and TNF/Fas pathways. We compared the baseline mRNA expression of these genes in sensitive versus resistant cell lines from the same disease type as well as between treated versus non-treated cells. The fold change of each gene at baseline (ratio sensitive/resistant) or 6 h after ASNase treatment (ratio treated/non-treated) were summarized in Tables 1 and 2. Genes whose expression was not detectable or did not vary in response to ASNase were not included.

For B-ALL cell lines, 26 genes (20 pro- and 6 anti-apoptotic) were more expressed in sensitive RS(4, 11) cells at baseline and 9 genes (7 pro and 2 anti-apoptotic) were less expressed, compared to resistant Nalm-6 cells. In ASNase-treated sensitive RS(4, 11) cells, the expression of 21 genes (16 pro- and 5 anti-apoptotic ) were induced compared to non-treated cells, whereas in ASNase-treated resistant Nalm-6 cells, only 5 pro-apoptotic genes were induced. These data indicate that the expression pattern of apoptosis genes is consistent with the phenotype of ASNase sensitivity/resistance of the analyzed cell lines. Moreover, in sensitive RS(4, 11) cells, 9 NFκB target genes (7 pro-apoptotic: *CASP4*, *PYCARD*, *RELB*, *NFκB2*, *BCL3*, *TNFSF10*, *FAS*; 2 anti-apoptotic: *BCL-2*, *BCL2A1*) were more expressed at baseline and 10 NFκB target genes (8 pro-apoptotic: *RELB*, *NFκB2*, *BCL3*, *NFκB1*, *TNF*, *TNFRSF1B*, *LTB*, *LTA*; 2 anti-apoptotic: *BCL2A1*, *NFκBIA*) were increased in ASNase-treated cells. Among these genes, four (3 pro-apoptotic *RELB*, *NFκB2*, *BCL3*; 1 anti-apoptotic: *BCL2A1*) were both more expressed at baseline and induced after ASNase treatment (Table 1). Therefore, the expression of pro-apoptotic genes, especially NFκB pathway-related genes, was more abundant in sensitive RS(4, 11) cells at baseline and after exposure to ASNase, as compared to resistant Nalm-6 cells, suggesting that the high expression of apoptosis-inducing genes and the increased activity of the NFκB pathway may be associated with the higher susceptibility of the RS(4, 11) cell line to ASNase.

With respect to malignant NK cell lines, 34 genes (25 pro- and 9 anti-apoptotic) were less expressed in sensitive MEC04 cells at baseline, and 5 genes (4 pro- and 1 anti-apoptotic) were more expressed, compared to resistant KHYG1 cells. In response to ASNase, only the expression of 1 pro-apoptotic gene was increased in sensitive MEC04 cells, whereas 2 pro-apoptotic genes were decreased. In resistant KHYG1 cells, 4 genes (2 pro-apoptotic and 2 anti-apoptotic) were induced, and 5 pro-apoptotic genes displayed reduced expression levels after ASNase treatment (Table 2). Contrary to B-ALL cell lines, apoptotic gene expression profile of malignant NK cell lines does not correlate with their sensitivity/resistance phenotype, with a poor expression of pro-apoptotic genes in sensitive MEC04 cells at baseline and after exposure to ASNase, compared to resistant KHYG1 cells.

**Figure 4:**
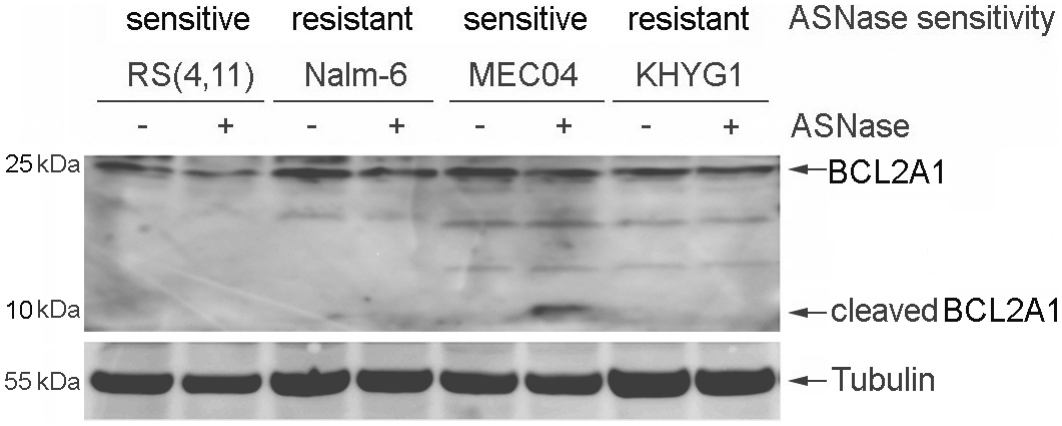
Analysis of BCL2A1 cleavage after exposure to ASNase. B-ALL (RS(4, 11), Nalm-6) and malignant NK (MEC04, KHYG1) cell lines were treated with 5 U/ml of pegylated-r-crisantaspase for 6 h. Non-treated cells served as controls. Protein expression was analyzed by Western Blot. All samples were loaded in the same gel.

We noticed that a specific anti-apoptotic member of the Bcl-2 family, *BCL2A1* (also known as *Bfl-1/A1*), was ~80 times more expressed at the transcript level in sensitive MEC04 cells, compared to resistant KHYG1 cells. Because the prosurvival BCL2A1 protein was recently reported to be converted into a pro-apoptotic factor after proteolytic cleavage^41^, we speculated that *BCL2A1* could represent a discriminating factor for ASNase susceptibility in these cell lines. As shown in Figure 4, the BCL2A1 protein was 1.7- fold more expressed in sensitive MEC04 cells at baseline, compared to resistant KHYG1 cells. Importantly, BCL2A1 cleavage was detected in sensitive MEC04 cells but not in resistant KHYG1 cells 6 h after ASNase treatment. These results suggest that BCL2A1 cleavage may be associated with the high sensitivity of MEC04 cells to ASNase and/or that the absence of BCL2A1 cleavage may be implicated in the resistance of KHYG1 cells (Figure 4).

## Discussion

ASNase is an important drug for ALL treatment. Although this molecule is efficient, some patients, especially adults, relapse, and refractory ALL cases are also reported. It is therefore essential to identify possible ASNase resistance determinants in leukemic cells. In this work, we sought to exploit the differential ASNase susceptibility of two pairs of B-ALL and malignant NK cell lines, in order to identify ASNase sensitivity/resistance-related genes or pathways, with a focus on classic compensatory responses and cell death under conditions of Asn/Gln limitation.

The survival of both highly ASNase-sensitive RS(4, 11) and MEC04 cell lines seems strictly dependent on extracellular Asn, because these two cell lines had undetectable protein expression of *AsnS* at baseline and after ASNase exposure, albeit showing high GlnS expression. Given that the phosphorylation of elF-2α was not induced (and even decreased) after ASNase exposure, AAR pathway was inactivated in both cell lines, which could be an explanation for the absence of induction of AsnS protein expression following ASNase treatment. Those observations are in line with previous findings showing absence of *AsnS* mRNA expression before and after ASNase treatment in sensitive RS(4, 11) cells^36^,^37^. It has been reported that the human B-cell leukemia cell lines, #1873 (presenting the same chromosomal translocation t(4, 11)(q21; q2311) as the RS(4, 11) cell line) and #1929, are Asn auxotrophic (meaning that they are unable to synthesize Asn by themselves). In these cell lines, *AsnS* gene is silenced and the promoter region of *AsnS* gene is highly methylated^42^,^43^. Therefore, in sensitive RS(4, 11) and MEC04 cell lines, absence of *AsnS* expression might be associated with specific epigenetic processes such as *AsnS* gene silencing.

In resistant KHYG1 cells, the high baseline expression of AsnS as well as the increase in both AsnS and GlnS expression following ASNase treatment could compensate for the deficiency in Asn and Gln by promoting the synthesis of both amino acids, explaining the low susceptibility of this cell line to ASNase. Yet, intriguingly, in ASNase-treated cells, the phosphorylation of Erk1/2 was greatly induced with a slight increase in AsnS expression, while phosphorylation of elF-2α was reduced to undetectable levels. These data are in contradiction with previous published data suggesting the existence of a positive regulatory loop between the AAR and MEK/ERK pathways as well as the involvement of the activated AAR pathway in the activation of the MEK/ERK pathway and in the induction of AsnS expression in response to amino acid restriction^27^,^28^. The involvement of the MEK/ERK pathway in the low ASNase-susceptibility of KHYG1 cell line will have to be further assessed by evaluating the changes in ASNase sensitivity of this cell line after ASNase treatment in presence of inhibitors of the MEK/ERK pathway. Interestingly, the resistant KHYG1 cell line carries a point mutation of p53 in codon 248. The role of this mutation is currently unknown^44^. Scian et al^45^,^46^ have reported that stable overexpression of two other p53 mutants, p53-D281G and p53-R273H mutants (corresponding to forms mutated in codon 281 and 273, respectively), can activate in the H1299 cell line the transcription of *AsnS* by binding to its promoter, resulting in an increase in the protein expression of *AsnS*. The authors propose that tumor-derived p53 mutants induce oncogenesis by transactivating growth-promoting genes such as *AsnS* and *hTERT* genes^45^,^46^. Hence, it will be interesting to test whether in the resistant KHYG1 cell line, mutated p53 (at codon 248) could positively modulate the expression of AsnS, representing another mechanism of ASNase resistance.

We demonstrated that ASNase-induced cell death was mediated by apoptosis, in agreement with previous reports^47^,^48^, and that the induction of apoptosis was expectedly delayed in resistant Nalm-6 and KHYG1 cell lines. In ASNase-treated resistant KHYG1 cells, the delayed induction of apoptosis is consistent with the classic compensatory mechanisms triggered after exposure to ASNase^10^,^28^. In sensitive MEC04 cells, our data suggest that pro-apoptotic cleavage of BCL2A1 may partly contribute to high ASNase sensitivity. Identification of candidate protease(s) activated in response to ASNase treatment and that could efficiently process the survival protein BCL2A1 to convert it into a cytotoxic factor is currently underway. The high sensitivity of RS(4, 11) cell line to ASNase could be associated with the imbalanced expression of pro- and anti- apoptotic genes, especially NFκB pathway-related genes, at baseline and after treatment, which may favor apoptosis in response to ASNase. Interestingly, in resistant Nalm-6 cell line, AsnS expression was low before and after exposure to ASNase in spite of the activation of the AAR pathway. The weak but detectable expression of AsnS in Nalm-6 cells may not be sufficient to explain the low susceptibility of this cell line to ASNase and other mechanisms including lack of induction of pro-apoptotic genes, especially NFκB target genes, at baseline and after exposure to ASNase may be involved.

In conclusion, our data provide evidence that, for B-ALL cell lines, changes in the expression of apoptosis-regulatory genes, especially NFκB-related genes, rather than AsnS-regulatory pathways could be crucial for the susceptibility of cells to ASNase. Regarding malignant NK cell lines, AsnS-regulatory pathways appear to be the main determinant implicated in the susceptibility of cells to ASNase, BCL2LA1 cleavage representing another potential mechanism. Anomalies making cells resistant to ASNase could be multiple and variable from one patient sample to another according to the type of disease and cellular context. Thus, our study provided a piece of puzzle for the understanding of ASNase-resistance mechanisms and for further development of more adaptive and personalized combinatory treatment for ALL (e.g. including inhibitors of Erk1/2 or modulators of the NFκB pathway).

## Methods

### Cell culture

The B-ALL cell lines RS(4, 11) (ATCC CRL-1873) and Nalm-6 (DSMZ ACC128) were cultured in RPMI 1640 medium (PAA laboratories, Mureaux, France), containing 10% fetal calf serum (FCS), 2 mM L-glutamine, 100 U penicillin and 100 μg streptomycin, at 37°C in a humidified atmosphere with 5% CO_2_. Malignant NK cell lines, MEC04 (nasal type NK T-cell lymphoma) and KHYG1 (aggressive NK cell leukemia), were cultured under the same condition in the presence of 10 ng/ml interleukin-2 (IL-2) (R&D system, Lille, France).

### Western Blot

Western blot was carried out as previously described^49^. The primary antibodies used were mouse monoclonal anti-glutamine synthetase (Abcam, Cambridge, UK), anti-β-actin antibody (Sigma-Aldrich, Saint-Louis, Missouri, USA), anti-α-tubulin antibody (Santa Cruz, Dallas, Texas USA.), rabbit polyclonal anti-asparagine synthetase (Abcam), anti-4E-BP1, anti-4E-BP1-pT70, anti-Erk1/2, anti-Erk1/2-pT202/Y204, anti-elF-2α-pS51, anti-elF-2α (Ozyme, Saint Quentin Yvelines, France), and anti-BCL2A1 antibody (Abcam). Secondary antibodies were as follows: biotinylated rabbit-anti mouse (Dako, Courtaboeuf, France) and biotinylated goat anti-rabbit (Invitrogen, Carlsbad, CA, USA). Detection was performed using Luminata Forte Western HRP substrate (Millipore Corporation, Billerica, MA). Quantifications were carried out by densitometric analysis using Kodak software as previously described^49^. β-actin or α-tubulin was used as loading control.

### Apoptosis assay

Cells were seeded at 0.2 × 10^6^ cells/ml in round-bottom 96-well plates and treated with increasing doses of pegylated r-crisantaspase^40^ from 5 × 10^−5^ U/ml to 5 U/ml for 24, 48 and 72 h. Non-treated cells were used as control. All samples were then subjected to Annexin V/PI apoptosis assay. Cells were incubated in 100 μl of Annexin V-binding buffer containing 10 μg/ml of PI (Sigma-Aldrich) and 2.5 μl Annexin V (Becton Dickinson, New Jersey, USA) for 15 min at room temperature before analysis by flow cytometry (Becton Dickinson, LSR II).

### TaqMan Low Density Array

Cells were seeded at 0.2 × 10^6^ cells/ml and treated with or without 5 U/ml of pegylated r-crisantaspase for 6 h. Total RNA was extracted using Trizol solution and RNA quality was assessed by Bioanalyser (Agilent technique, Les Ulis, France). The reverse transcription was performed using High Capacity RNA-to-cDNA Master Mix (Life Technologies, Carlsbad, CA, USA) with 1 μg of total RNA in a volume of 20 μl. The product of reverse transcription diluted in TaqMan® Universal PCR Master Mix (Life Technologies) was then loaded into TaqMan® Human Apoptosis Array (Life Technologies) and quantitative PCR was performed with the Applied Biosystems 7900 Sequence Detection system (Life Technologies) according to the manufacturer's instructions. Relative quantification (RQ) was obtained using the equation RQ = 2^−ΔΔCt^ after normalization of the C_t_ data to that of the reference gene, where C_t_ is the threshold cycle of fluorescence detection. Results were the combined data of two independent experiments. The reference gene for baseline expression analysis was *18S*. The mean and standard deviation (S.D.) were Ct = 9.8 ± 0.4 for non-treated B-ALL samples and Ct = 10.2 ± 0.5 for non-treated malignant NK samples. *18S*, *GADPH* and *β-actin* were the reference genes for normalizing data of ASNase-treated samples to that of non-treated samples. RS(4, 11) cells: *18S* Ct = 8.7 ± 0.1; *GADPH* Ct = 17.5 ± 0.1; *β-actin* Ct = 17.9 ± 0.1. Nalm-6 cells: *18S* Ct = 8.4 ± 0.2; *GADPH* Ct = 17.9 ± 0.1; *β-actin* Ct = 18.4 ± 0.1. MEC04: *18S* Ct = 8.6 ± 0.2; *GADPH* Ct = 16.7 ± 0.2; *β-actin* Ct = 18.9 ± 0.2. KHYG1: *18S* Ct = 9.9 ± 0.3; *GADPH* Ct = 17.4 ± 0.1; *β-actin* Ct = 17.3 ± 0.2.

## Figures and Tables

**Table 1:** Expression of apoptosis-regulatory genes in B-ALL cell lines at baseline and in response to ASNase

		Baseline expression		RS(4, 11)		Nalm-6	
Genes		RS(4, 11)/Nalm-6		ASNase+/ASNase−		ASNase+/ASNase−	
**Pro-apoptotic**		mean	SD	mean	SD	mean	SD
BCL-2 family	BID	3,43	0,48	0,80	0,06	0,39	0,05
	BAD	2,71	0,47	1,32	0,15	0,67	0,10
	PMAIP1	2,48	0,71	2,34	0,47	2,21	0,18
	BBC3	0,82	0,14	5,99	1,18	1,21	0,10
	HRK	Ct ≥ 35/Ct = 31 (≤0,29)		ND		2,15	0,25
	BNIP3	Ct ≥ 35/Ct = 24 (≤0,002)		ND		0,96	0,12
Caspase-related	BCAP31	6,70	1,19	0,87	0,07	0,81	0,09
	ESRRBL1	5,90	1,12	0,97	0,15	0,96	0,07
	CASP5	4,16	2,92	1,28	0,66	5,77	1,09
	CASP4	2,55	0,27	0,99	0,15	0,92	0,07
	NALP1	2,26	0,36	0,94	0,15	0,80	0,14
	PYCARD	2,10	0,28	0,72	0,06	0,59	0,05
	HIP1	0,57	0,10	2,07	0,49	0,99	0,08
	CASP6	0,40	0,001	0,83	0,22	0,90	0,10
NFkB-related	CARD9	13,24	4,26	2,06	0,30	1,28	0,17
	RELB	9,42	0,17	4,22	0,66	1,34	0,20
	NFKB2	3,58	0,40	4,26	0,44	1,27	0,11
	IKBKG	3,23	0,17	1,22	0,11	1,03	0,08
	BCL3	2,49	0,83	7,71	0,70	3,11	0,90
	IKBKB	2,16	0,52	1,28	0,13	1,07	0,08
	NFKB1	1,55	0,16	2,11	0,16	0,85	0,06
	REL	0,65	0,18	2,52	0,31	1,35	0,15
	CARD4	0,15	0,001	1,08	0,08	0,64	0,05
TNF/Fas-realted	TNFSF10	Ct = 31,1/Ct > 35 (≥23,38)		1,07	0,15	ND	
	TNFRSF25	17,18	3,79	1,22	0,14	0,59	0,06
	FAS	3,71	0,82	0,90	0,21	0,59	0,05
	TNFRSF1A	2,68	0,37	2,35	0,31	2,22	0,31
	LRDD	2,64	0,47	1,40	0,10	1,17	0,09
	TNF	1,61	0,43	3,90	0,29	1,36	0,13
	TNFRSF1B	1,48	0,02	2,36	0,24	1,11	0,09
	TNFRSF10A	Ct = 34,4/Ct ≥ 35		6,07	0,83	ND	
	LTB	0,51	0,01	3,04	0,28	0,90	0,07
	LTA	0,40	0,12	3,44	0,27	1,28	0,10
	TNFRSF21	0,25	0,02	0,59	0,05	0,42	0,04
	TNFRSF10B	0,05	0,02	5,61	1,25	1,04	0,09
**Anti-apoptotic**							
BCL-2 family	BCL2	10,70	2,04	1,44	0,19	0,94	0,08
	BCL2A1	2,91	0,39	2,64	0,34	1,55	0,52
	NFKBIA	1,98	0,48	2,43	0,43	1,28	0,14
	TA-NFKBH	0,48	0,13	4,96	0,67	1,29	0,13
TNF/Fas-realted	BIRC2	2,82	1,22	1,71	0,76	1,87	0,21
	BIRC3	Ct = 31,5/Ct ≥ 35 (≥14,45)		10,99	0,95	ND	
	BIRC7	Ct = 32,7/Ct ≥ 35 (≥4,66)		0,62	0,42	ND	
	BIRC4	2,02	0,30	1,43	0,19	0,95	0,07
	BIRC1	0,29	0,02	2,44	0,18	1,44	0,29

The cut-off for more rich or increased expression (Red) was 2-fold and the cut-off for low or decreased expression (Green) was 0.5-fold. NFkB target genes are marked in Blue. SD = Standard deviation; Ct = the threshold cycle of fluorescence detection; ND = Non-detectable (Ct ≥ 35).

**Table 2:** Expression of apoptosis regulatory genes in malignant NK cell lines at baseline and in response to ASNase

		Baseline expression		MEC04		KHYG1	
Genes		MEC04/KHYG1		ASNase+/ASNase−		ASNase+/ASNase−	
**Pro-apoptotic**		mean	SD	mean	SD	mean	SD
BCL-2 family	BBC3	0,75	0,03	9,46	2,76	4,84	1,05
	BAD	0,48	0,05	1,10	0,20	0,93	0,24
	BAK1	0,45	0,03	0,68	0,16	1,07	0,21
	BCL2L11	0,25	0,02	0,94	0,18	1,17	0,25
	BID	0,22	0,09	0,51	0,09	0,62	0,17
Caspase-related	CASP9	2,01	0,08	1,26	0,37	1,02	0,22
	CASP10	2,00	0,01	1,46	0,31	1,54	0,31
	CASP8	1,20	0,18	1,52	0,34	2,40	0,48
	CASP3	0,45	0,16	0,65	0,12	0,71	0,20
	CASP1	0,44	0,21	0,99	0,19	0,95	0,29
	HIP1	0,17	0,05	1,02	0,18	0,87	0,20
	ESRRBL1	0,04	0,02	1,00	0,47	1,30	0,90
NFkB-related	PYCARD	Ct ≥ 35/Ct = 23,2 (≤0,001)		ND		0,82	0,17
	CARD6	6,80	0,25	1,64	0,65	0,83	0,23
	RIPK2	4,78	1,61	0,97	0,25	0,64	0,21
	IKBKG	0,45	0,01	1,13	0,21	0,92	0,18
	CARD4	0,41	0,05	1,10	0,21	1,21	0,25
	IKBKB	0,34	0,06	1,30	0,25	0,96	0,20
	NFKB1	0,19	0,03	1,10	0,22	0,68	0,18
	RELB	0,11	0,01	1,67	0,34	1,17	0,28
	NFKB2	0,10	0,01	1,54	0,27	0,96	0,19
TNF/Fas-realted	DEDD	0,49	0,06	0,74	0,15	0,87	0,18
	PEA15	0,44	0,14	0,83	0,16	0,90	0,20
	DEDD2	0,43	0,02	1,46	0,27	1,17	0,25
	TNFRSF1B	0,29	0,00	0,59	0,11	0,39	0,08
	FASLG	0,28	0,03	0,44	0,10	0,54	0,11
	TNFSF10	0,14	0,06	0,53	0,11	0,23	0,08
	TNFRSF10B	0,06	0,002	1,34	0,25	1,32	0,28
	LTB	0,02	0,001	0,87	0,42	0,28	0,09
	TNF	0,004	0,001	0,44	0,18	0,32	0,08
	LTA	0,002	0,001	0,69	0,19	0,14	0,04
**Anti-apoptotic**							
BCL-2 family	BCL2A1	80,79	46,98	1,67	0,31	1,00	0,49
	BCL2L1	0,43	0,05	0,90	0,28	0,83	0,24
	BCL2	0,31	0,11	0,93	0,17	0,89	0,20
Caspase-related	ICEBERG	Ct ≥ 35/Ct = 32 (≤0,22)		ND		3,87	0,80
NFkB-related	TA-NFKBH	0,50	0,07	0,63	0,11	1,27	0,26
	NFKBIB	0,46	0,04	1,00	0,20	0,97	0,19
	NFKBIE	0,27	0,02	0,63	0,12	0,82	0,16
	NFKBIA	0,10	0,01	1,84	0,33	1,03	0,21
TNF/Fas-realted	BIRC1	2,07	1,27	1,66	0,30	3,45	0,79
	BIRC3	0,10	0,03	1,07	0,19	0,53	0,13
	BIRC7	Ct ≥ 35/Ct = 30,7 (≤0,04)		ND		0,90	0,18

The cut-off for more rich or increased expression (Red) was 2-fold and the cut-off for low or decreased expression (Green) was 0.5-fold. NFkB target genes are marked in Blue. SD = Standard deviation; Ct = the threshold cycle of fluorescence detection; ND = Non-detectable (Ct ≥ 35).

## References

[b1] Pieters R. *et al.* L-asparaginase treatment in acute lymphoblastic leukemia: a focus on Erwinia asparaginase. Cancer 117, 238–249 (2011). 20824725 10.1002/cncr.25489 PMC3000881

[b2] Avramis V. I. Asparaginases: a successful class of drugs against leukemias and lymphomas. J Pediatr Hematol Oncol 33, 573–579 (2011). 22042273 10.1097/MPH.0b013e31823313be

[b3] Yong W. *et al.* L-asparaginase-based regimen in the treatment of refractory midline nasal/nasal-type T/NK-cell lymphoma. International journal of hematology 78, 163–167 (2003). 12953813 10.1007/BF02983387

[b4] Yamaguchi M. *et al.* Phase I study of dexamethasone, methotrexate, ifosfamide, L-asparaginase, and etoposide (SMILE) chemotherapy for advanced-stage, relapsed or refractory extranodal natural killer (NK)/T-cell lymphoma and leukemia. Cancer science 99, 1016–1020, 10.1111/j.1349-7006.2008.00768.x (2008). 18294294 PMC11158592

[b5] Jaccard A. *et al.* L-asparaginase-based treatment of 15 western patients with extranodal NK/T-cell lymphoma and leukemia and a review of the literature. Annals of oncology: official journal of the European Society for Medical Oncology/ESMO 20, 110–116, 10.1093/annonc/mdn542 (2009). 18701429

[b6] Asselin B. L. *et al.* In vitro and in vivo killing of acute lymphoblastic leukemia cells by L-asparaginase. Cancer Res 49, 4363–4368 (1989). 2743326

[b7] Avramis V. I. & Tiwari P. N. Asparaginase (native ASNase or pegylated ASNase) in the treatment of acute lymphoblastic leukemia. Int J Nanomedicine 1, 241–254 (2006). 17717965 PMC2426805

[b8] Haskell C. M. & Canellos G. P. l-asparaginase resistance in human leukemia--asparagine synthetase. Biochem Pharmacol 18, 2578–2580 (1969). 4935103 10.1016/0006-2952(69)90375-x

[b9] Hutson R. G. *et al.* Amino acid control of asparagine synthetase: relation to asparaginase resistance in human leukemia cells. The American journal of physiology 272, C1691–1699 (1997). 9176161 10.1152/ajpcell.1997.272.5.C1691

[b10] Richards N. G. & Kilberg M. S. Asparagine synthetase chemotherapy. Annu Rev Biochem 75, 629–654 (2006). 16756505 10.1146/annurev.biochem.75.103004.142520 PMC3587692

[b11] Aslanian A. M., Fletcher B. S. & Kilberg M. S. Asparagine synthetase expression alone is sufficient to induce l-asparaginase resistance in MOLT-4 human leukaemia cells. Biochem J 357, 321–328 (2001). 11415466 10.1042/0264-6021:3570321 PMC1221958

[b12] Panosyan E. H. *et al.* Deamination of glutamine is a prerequisite for optimal asparagine deamination by asparaginases in vivo (CCG-1961). Anticancer Res 24, 1121–1125 (2004). 15154634

[b13] Rotoli B. M. *et al.* Inhibition of glutamine synthetase triggers apoptosis in asparaginase-resistant cells. Cell Physiol Biochem 15, 281–292 (2005). 16037693 10.1159/000087238

[b14] Tardito S. *et al.* The inhibition of glutamine synthetase sensitizes human sarcoma cells to L-asparaginase. Cancer Chemother Pharmacol 60, 751–758 (2007). 17256128 10.1007/s00280-007-0421-z

[b15] Offman M. N. *et al.* Rational engineering of L-asparaginase reveals importance of dual activity for cancer cell toxicity. Blood 117, 1614–1621, 10.1182/blood-2010-07-298422 (2011). 21106986

[b16] Andrulis I. L., Argonza R. & Cairney A. E. Molecular and genetic characterization of human cell lines resistant to L-asparaginase and albizziin. Somatic cell and molecular genetics 16, 59–65 (1990). 1968681 10.1007/BF01650480

[b17] Aslanian A. M. & Kilberg M. S. Multiple adaptive mechanisms affect asparagine synthetase substrate availability in asparaginase-resistant MOLT-4 human leukaemia cells. Biochem J 358, 59–67 (2001). 11485552 10.1042/0264-6021:3580059 PMC1222032

[b18] Avramis V. I. Asparaginases: biochemical pharmacology and modes of drug resistance. Anticancer Res 32, 2423–2437 (2012). 22753699

[b19] Gutierrez J. A. *et al.* An inhibitor of human asparagine synthetase suppresses proliferation of an L-asparaginase-resistant leukemia cell line. Chem Biol 13, 1339–1347 (2006). 17185229 10.1016/j.chembiol.2006.10.010 PMC3608209

[b20] Li B. S. *et al.* The downregulation of asparagine synthetase expression can increase the sensitivity of cells resistant to l-asparaginase. Leukemia 20, 2199–2201, 10.1038/sj.leu.2404423 (2006). 17039232

[b21] Ikeuchi H. *et al.* A sulfoximine-based inhibitor of human asparagine synthetase kills L-asparaginase-resistant leukemia cells. Bioorganic & medicinal chemistry 20, 5915–5927, 10.1016/j.bmc.2012.07.047 (2012). 22951255

[b22] Ramirez M. *et al.* Mutations activating the yeast eIF-2 alpha kinase GCN2: isolation of alleles altering the domain related to histidyl-tRNA synthetases. Molecular and cellular biology 12, 5801–5815 (1992). 1448107 10.1128/mcb.12.12.5801-5815.1992 PMC360520

[b23] Harding H. P. *et al.* Regulated translation initiation controls stress-induced gene expression in mammalian cells. Mol Cell 6, 1099–1108 (2000). 11106749 10.1016/s1097-2765(00)00108-8

[b24] Harding H. P. *et al.* An integrated stress response regulates amino acid metabolism and resistance to oxidative stress. Mol Cell 11, 619–633 (2003). 12667446 10.1016/s1097-2765(03)00105-9

[b25] Ye J. *et al.* The GCN2-ATF4 pathway is critical for tumour cell survival and proliferation in response to nutrient deprivation. Embo J 29, 2082–2096, 10.1038/emboj.2010.81 (2010). 20473272 PMC2892366

[b26] Balasubramanian M. N., Butterworth E. A. & Kilberg M. S. Asparagine synthetase: regulation by cell stress and involvement in tumor biology. American journal of physiology. Endocrinology and metabolism 304, E789–799, 10.1152/ajpendo.00015.2013 (2013). 23403946 PMC3625782

[b27] Gallinetti J., Harputlugil E. & Mitchell J. R. Amino acid sensing in dietary-restriction-mediated longevity: roles of signal-transducing kinases GCN2 and TOR. Biochem J 449, 1–10, 10.1042/BJ20121098 (2013). 23216249 PMC3695616

[b28] Thiaville M. M. *et al.* MEK signaling is required for phosphorylation of eIF2alpha following amino acid limitation of HepG2 human hepatoma cells. J Biol Chem 283, 10848–10857, 10.1074/jbc.M708320200 (2008). 18287093 PMC2447663

[b29] Nobukuni T. *et al.* Amino acids mediate mTOR/raptor signaling through activation of class 3 phosphatidylinositol 3OH-kinase. Proc Natl Acad Sci U S A 102, 14238–14243 (2005). 16176982 10.1073/pnas.0506925102 PMC1242323

[b30] Reiling J. H. & Sabatini D. M. Stress and mTORture signaling. Oncogene 25, 6373–6383, 10.1038/sj.onc.1209889 (2006). 17041623

[b31] Proud C. G. Amino acids and mTOR signalling in anabolic function. Biochem Soc Trans 35, 1187–1190, 10.1042/BST0351187 (2007). 17956308

[b32] Yamaguchi S. *et al.* ATF4-mediated induction of 4E-BP1 contributes to pancreatic beta cell survival under endoplasmic reticulum stress. Cell metabolism 7, 269–276, 10.1016/j.cmet.2008.01.008 (2008). 18316032

[b33] Whitney M. L., Jefferson L. S. & Kimball S. R. ATF4 is necessary and sufficient for ER stress-induced upregulation of REDD1 expression. Biochemical and biophysical research communications 379, 451–455, 10.1016/j.bbrc.2008.12.079 (2009). 19114033 PMC2656673

[b34] Bunpo P. *et al.* GCN2 protein kinase is required to activate amino acid deprivation responses in mice treated with the anti-cancer agent L-asparaginase. J Biol Chem 284, 32742–32749, 10.1074/jbc.M109.047910 (2009). 19783659 PMC2781691

[b35] Stams W. A. *et al.* Sensitivity to L-asparaginase is not associated with expression levels of asparagine synthetase in t(12;21)+ pediatric ALL. Blood 101, 2743–2747, 10.1182/blood-2002-08-2446 (2003). 12433682

[b36] Fine B. M., Kaspers G. J., Ho M., Loonen A. H. & Boxer L. M. A genome-wide view of the in vitro response to l-asparaginase in acute lymphoblastic leukemia. Cancer Res 65, 291–299 (2005). 15665306

[b37] Hermanova I., Zaliova M., Trka J. & Starkova J. Low expression of asparagine synthetase in lymphoid blasts precludes its role in sensitivity to L-asparaginase. Experimental hematology 40, 657–665, 10.1016/j.exphem.2012.04.005 (2012). 22542578

[b38] Stams W. A. *et al.* Asparagine synthetase expression is linked with L-asparaginase resistance in TEL-AML1-negative but not TEL-AML1-positive pediatric acute lymphoblastic leukemia. Blood 105, 4223–4225 (2005). 15718422 10.1182/blood-2004-10-3892

[b39] Holleman A. *et al.* Gene-expression patterns in drug-resistant acute lymphoblastic leukemia cells and response to treatment. N Engl J Med 351, 533–542 (2004). 15295046 10.1056/NEJMoa033513

[b40] Chien W. W. *et al.* Pharmacology, immunogenicity, and efficacy of a novel pegylated recombinant Erwinia chrysanthemi-derived L-asparaginase. Investigational new drugs 10.1007/s10637-014-0102-9 (2014). 24829072

[b41] Valero J. G. *et al.* micro-Calpain conversion of antiapoptotic Bfl-1 (BCL2A1) into a prodeath factor reveals two distinct alpha-helices inducing mitochondria-mediated apoptosis. PloS one 7, e38620, 10.1371/journal.pone.0038620 (2012). 22745672 PMC3379997

[b42] Ren Y., Roy S., Ding Y., Iqbal J. & Broome J. D. Methylation of the asparagine synthetase promoter in human leukemic cell lines is associated with a specific methyl binding protein. Oncogene 23, 3953–3961, 10.1038/sj.onc.1207498 (2004). 15048083

[b43] Ding Y., Li Z. & Broome J. D. Epigenetic changes in the repression and induction of asparagine synthetase in human leukemic cell lines. Leukemia 19, 420–426, 10.1038/sj.leu.2403639 (2005). 15674423

[b44] Yagita M. *et al.* A novel natural killer cell line (KHYG-1) from a patient with aggressive natural killer cell leukemia carrying a p53 point mutation. Leukemia 14, 922–930 (2000). 10803526 10.1038/sj.leu.2401769

[b45] Scian M. J. *et al.* Tumor-derived p53 mutants induce oncogenesis by transactivating growth-promoting genes. Oncogene 23, 4430–4443, 10.1038/sj.onc.1207553 (2004). 15077194

[b46] Scian M. J. *et al.* Modulation of gene expression by tumor-derived p53 mutants. Cancer Res 64, 7447–7454, 10.1158/0008-5472.CAN-04-1568 (2004). 15492269

[b47] Ando M. *et al.* Selective apoptosis of natural killer-cell tumours by l-asparaginase. Br J Haematol 130, 860–868 (2005). 16156856 10.1111/j.1365-2141.2005.05694.x

[b48] Suto H. *et al.* Suppression of eIF4E expression by L-Asparaginase. Acta Haematol 123, 215–219 (2010). 20424434 10.1159/000313362

[b49] Chien W. W. *et al.* Cyclin-dependent kinase 1 expression is inhibited by p16(INK4a) at the post-transcriptional level through the microRNA pathway. Oncogene 30, 1880–1891, 10.1038/onc.2010.570 (2011). 21170085

